# Synthesis and Chemical Functionalization of Pseudo-Homogeneous Catalysts for Biodiesel Production—Oligocat

**DOI:** 10.3390/polym14010019

**Published:** 2021-12-22

**Authors:** Vitor Vlnieska, Aline S. Muniz, Angelo R. S. Oliveira, Maria A. F. César-Oliveira, Danays Kunka

**Affiliations:** 1 Federal University of Paraná (UFPR), Rua Coronel Francisco Heráclito dos Santos, 100, Jardim das Américas, Curitiba 81531-980, PR, Brazil; mafco@quimica.ufpr.br (A.S.M.); arso@ufpr.br (A.R.S.O.); mafco@ufpr.br (M.A.F.C.-O.); 2 EMPA—Swiss Federal Laboratories for Materials Science & Technology, Überlandstrasse 129, 8600 Dübendorf, Switzerland; 3 Karlsruhe Institute of Technology (KIT), Institute of Microstructure Technology, Hermann-von-Helmholtz-Platz 1, 76344 Eggenstein-Leopoldshafen, Germany; danays.kunka@kit.edu

**Keywords:** biodiesel, second generation feedstock, aromatic hydroxy acids, polyester, oligoester, sulfonation, acid catalyst, GPC, ^1^H NMR, TGA

## Abstract

With the increase in global demand for biodiesel, first generation feedstock has drawn the attention of governmental institutions due to the correlation with large land farming areas. The second and third feedstock generations are greener feedstock sources, nevertheless, they require different catalytic conditions if compared with first generation feedstock. In this work, we present the synthesis and characterization of oligoesters matrices and their functionalization to act as a pseudo-homogeneous acid catalyst for biodiesel production, named Oligocat. The main advantage of Oligocat is given due to its reactional medium interaction. Initially, oligocat is a solid catalyst soluble in the alcoholic phase, acting as a homogeneous catalyst, providing better mass transfer of the catalytic groups to the reaction medium, and as the course of the reaction happens, Oligocat migrates to the glycerol phase, also providing the advantage of easy separation of the biodiesel. Oligocat was synthesized through polymerization of aromatic hydroxy acids, followed by a chemical functionalization applying the sulfonation technique. Characterization of the catalysts was carried out by infrared spectroscopy (FTIR), nuclear magnetic resonance spectroscopy (NMR), gel permeation chromatography (GPC), and thermogravimetric analysis (TGA). The synthesized oligomers presented 5357 g·mol^−1^ (Mw) and 3909 g·mol^−1^ (Mn), with a moderate thermal resistance of approximately 175 °C. By sulfonation reaction, it was possible to obtain a high content of sulphonic groups of nearly 70 mol%, which provided the catalytic activity to the oligomeric matrix. With the mentioned physical–chemical properties, Oligocat is chemically designed to convert second generation feedstock to biodiesel efficiently. Preliminary investigation using Oligocat for biodiesel production resulted in conversion rates higher than 96.5 wt.%.

## 1. Introduction

### 1.1. Biodiesel in Brief Facts

Environmentally friendly and renewable energy sources are one of the alternatives to control and reduce global warming and climate change. In this context, biodiesel is a decisive renewable energy source to replace petrol diesel [1]. Regarding biodiesel demand, for example, since the 2000 decade, the production of biodiesel in Germany has increased by almost seventy-fold [2], reaching approximately 2.35 × 10^3^ (Tons·year^−1^). With the increase of biodiesel production, first generation feedstock has drawn the attention of several governmental institutions due to its direct correlation with large land farming areas. Estimations indicate that farming areas equally as large as The Netherlands was exploited to deliver first generation feedstock to biodiesel production [2,3]. In this case, if one considers the entire life-cycle greenhouse gas emissions, biodiesel produced from first generation feedstock generates higher CO_2_ emission than petroleum diesel [3]. Nevertheless, biodiesel can be definitely a greener energy source than petroleum, and an efficient approach to reduce CO_2_ emission levels is to produce it from second generation feedstock [4].

Second generation feedstock is defined as any triacylglycerol source (TAG’s) with low commercial value [4,5,6,7]. As the second generation feedstock presents significant amounts of H_2_O and free fatty acids (FFA) in its composition, basic catalysis becomes impracticable due to the side products formed with these feedstock contaminants [3]. To efficiently convert second generation feedstock to biodiesel, one might consider acid catalysis, which has the advantage of not being affected by side products generated from water and FFA contents in the feedstock matrix. Nonetheless, acid catalysis presents the disadvantage of low kinetic velocity, being circa four thousand-times slower than basic catalysis [8].

Independently of the reaction medium, catalysts can be synthesized as either being a homogeneous or heterogeneous material. For biodiesel production, a homogeneous catalyst is a chemical compound soluble in both of the reaction medium liquid phases, which are the lipidic (TAG’s) or alcoholic phases. Consequently, one defines a catalyst as heterogeneous if the chemical compound is not soluble at these phases. Heterogeneous catalysts present the advantage of easy separation and recovery from the lipidic and alcoholic phases. Nevertheless, the mass transfer properties of the catalytic group are poorer, generating longer times for reaction. On the other hand, homogeneous catalysis promotes efficient mass transfer of the catalytic groups, although contamination and difficulties to completely remove the catalyst from the final product are usual challenges in biodiesel production [1,7,9,10,11].

Here, we present the synthesis and characterization of our designed acid catalyst, named Oligocat. Initially, Oligocat acts as a homogeneous catalyst, providing better mass transfer of the catalytic groups to the reaction medium, and as the course of the reaction happens, Oligocat migrates to the glycerol phase, also providing the advantage of easy separation of the biodiesel. A deeper investigation of Oligocat catalytic efficiency is presented by Vlnieska et al. 2021 [3], where one can note a conversion efficiency higher than 96.5 wt.% within one reaction cycle, which was achieved after optimizing biodiesel reaction parameters. To better understand how Oligocat was designed, the main characteristics of this catalyst are once more presented:

- to be applied in second generation stock;
- being a Brønsted–Lowry acid catalyst;
- to act initially as homogeneous catalyst;
- to provide conversion yield of TAG’s to FA(X)E above 96.5 wt.% within one reaction cycle.

### 1.2. Biodiesel Heterogeneous Catalysts

When compared with homogenous catalysts, the main advantages of heterogeneous catalysts are an easier separation from biodiesel and as well as its reusability in several reaction cycles. Liquid homogeneous acid catalysts can potentially harm the environment due to the usage of higher amounts of resources to purify the biodiesel phase after completion of the reaction. For example, the most applied liquid acid catalysts such as sulfuric acid and hydrochloric acid can also often lead to equipment corrosion [12]. On the other hand, heterogeneous solid acid catalysts significantly reduce the volume of resources for this step. As main disadvantages, due to the slower kinetics of acid catalysis, harder reaction conditions need to be employed in order to achieve the conversion rate determined by either EN 14214 or ASTM D6751 biodiesel standards. Also, the leaching process of the active groups into the biodiesel phases, which results in deactivation of the catalyst, is often a challenge for heterogeneous solid acid catalysts technology [13,14].

In the literature, almost limitless kinds of catalysts, either being acid or basic and homo- or heterogenous have been reported. Porous materials such as zeolites, with pore size varying from 2 to 50 nm (known also as mesoporous materials), ionic resins, and inorganic acids are the most used in acid catalysis. On the other side, alkali oxides are the most applied for basic catalysis, in which hybrid catalytic matrices can be found, for example, carbon extracted from date seed modified with Ca and Mg oxides, such as KOH-CaO, CaO, and hybrid CaO/Al_2_O_3_ aerogels have been studied [15,16]. Heteropoly acid derivatives (HPD) and bifunctional catalysts have been reported as acid catalytic matrices for biodeisel production [17,18]. Enzymatic catalysis have been studied through lipolysis reactions. Although conversion rates, selectivity, and recovery of immobilized enzymes being efficient, scalability for larger production volumes seems to be a challenge for this technology [19]. Investigation of nanocatalysts have been reported with satisfactory yields for biodiesel conversion, either by acid or basic catalytic mechanism reactions. For example, a solid acid nanocatalyst produced from TiO with propyl sulfonic acid was evaluated by Gardy J. et al. (2019), reaching a conversion rate of 98.3% of biodiesel from waste cooking oil [20]. As well, it is interesting to note that research made over the last decades for waste materials such as ashes from diverse origins, which have been explored as raw materials for catalysts in biodiesel production, have shown high conversion rates up to 96% being reported [21].

New approaches to catalyst preparation such as sulphonated oxides, for example, zirconia oxide (SO_4_^2−^/ZrO_2_) niobium (SO_4_^2−^/Nb_2_O_5_), ionic-liquid (IL) and polymeric ionic liquid (PIL), and sulphonated ionic liquid immobilized in metal-organic-frameworks (SIL-MOF) have been recently explored [22]. Ionic and polymeric ionic liquids have been investigated within several immobilization techniques because of the advantage of being easily separated from the biodiesel reaction medium thorough external magnetization [23], besides, they present high stability, excellent recovery, and reusability. Nevertheless, IL and PIL usually have high viscosity and low solubility in biodiesel phases, resulting in low mass transfer of the catalytic groups to the reaction medium. To tackle this challenge, one can profit from the immobilization of IL and PIL in nanoparticles, for example iron oxide (Fe_3_O_4_). This kind of immobilization is promising because it increases the catalyst surface contact area. Xie et al. (2021) applied grafting copolymerization technique to immobilize an IL in magnetic silica composite. Conversion rates of biodiesel up to 94.2 ± 2.7 were achieved, and minor yield losses were observed within five reaction cycles [23]. Zhang et al. (2017) reached 92.8% of biodiesel conversion applying PIL immobilized on a magnetic mesoporous carrier, the synthesized PIL remained with no significant catalytic activity losses after being recovered after five consecutive cycles [24].

Nonetheless, one has to consider that magnetic nanoparticles usually suffer from aggregation into large clusters, which results in non-uniform dispersion at the reaction medium, lowering the catalyst’s performance [12]. In order to tackle the drawbacks of solid catalyst technology in this work, we have synthesized a polymeric solid acid catalyst, named Oligocat, which is soluble in the alcoholic phase. This solubility property optimizes the mass transfer of the catalytic groups to the reaction medium. As the course of the reaction happens, Oligocat migrates to the glycerol phase, not contaminating the biodiesel, and is easily recovered and reutilized. With Oligocat, the generated biodiesel does not need to be purified or have the pH corrected. Properties and performance of Oligocat catalyst for biodiesel production is investigated in details by Vlnieska et al. 2021 [3].

### 1.3. Mass Polymerization and Sulfonation Reactions to Generate Oligocat

Oligocat is fundamentally synthesized in two reaction steps, using Winfield and Dickson thermopolymerization reaction followed by the sulfonation technique, which is responsible to insert catalytic groups into the polymeric chains. These two synthesis techniques are historically shortly presented.

**Figure 1:**
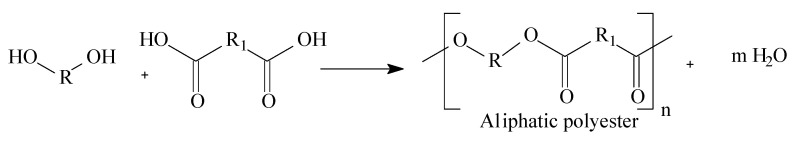
General aliphatic polyester chemical structure.

In 1930, Carothers and co-workers synthesized the first aliphatic polyester [25]. The structure of a polyester is generated by repetitive units linked by an ester function group (–COO–), which originates the polymer chains as presented in Figure 1:

In Figure 1, the letter “R” represents the diol carbonic chains and “R1” represents the acid carbonic chain. Letter “n” represents the number of monomers that form the polyester polymeric chain.

**Figure 2:**
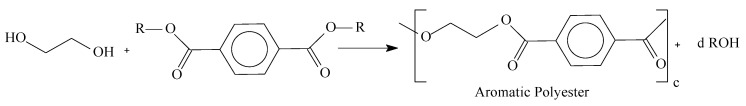
Transesterification reaction of a general aromatic diester and a diol.

Aliphatic polymers produced by Carothers had low melting point and were prone to hydrolysis. Most probably due to the poor properties of aliphatic polyesters, one decade later Whinfield and Dickson published the synthesis of an aromatic polyester denominated poly(ethylene terephthalate)—PET, which was obtained applying the synthesis technique proposed by Carothers, but this time using as reagent one aromatic diester. The outcome was a polymer with high melting point and high hydrolysis resistance, allowing its application in several areas of industry. After this discovery, several aromatic molecules were investigated and studied. Commercially, polymers PET—poly(ethylene terephthalate) and PBT—poly(butylene terephthalate) are the most relevant ones. They are applied in many areas such as synthetic fibers, films, packing, etc. Aromatic polyesters were introduced for industrial manufacturing by Imperial Chemistry (ICI) in 1949 and Du Pont in 1953, utilizing Whinfield and Dickinson, and Carothers synthesis techniques. Their synthesis claims that in general, a polyester can be obtained by condensation of a diacid with a diol (esterification) or through condensation of a diester with a diol (transesterification). As an example, the structure of poly (ethylene terephthalate)—PET and a generic aliphatic polymer are presented in Figure 2 [26,27].

In Figure 2: “R” represents the ester carbonic chains, “c” means the number of repetitive units from the polymeric chain, and letter “d” is the subproduct stoichiometric coefficient, which in this case is an aliphatic alcohol.

**Figure 3:**
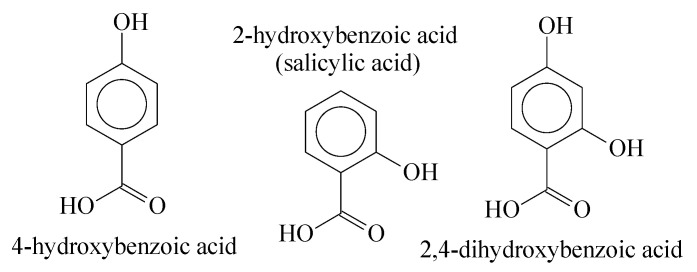
Aromatic molecules with hydroxyl and acid groups.

Esterification and transesterification are thermodynamically reversible reactions and due to the hydrolysation of the ester groups, degradation easily occurs with aliphatic structures. Nevertheless, as mentioned above, when an aromatic acid is applied as a condensation reagent, the solubility in water is dramatically reduced, making it difficult for the interaction of the water molecules with ester groups [26]. For this work, we have utilized aromatic compounds with phenol and acid groups in their structures to produce Oligocat, as Figure 3 presents.

The main advantage of using this kind of molecule is to promote the condensation (esterification) reaction within one single structure, as well as resulting in polymers with high hydrolysis resistance. Nevertheless, due to the mass polymerization technique itself, one might expect considerable variation of the physico-chemical properties and a non-complete control of polymeric chain reaction growth [28,29]. To convert polymer matrices to catalysts, one usually has to consider a chemical functionalization in the polymer chains, which aims to promote a catalysis cycle in a chemical reaction. Two other properties are of fundamental importance to be measured; first is the size of organic chains and second is its polarity in a broad range of chemical environments. If these two characteristics are known and controlled, one can synthesize polymers with specific catalytic properties for a specific class of chemical reactions. That said, functionalized polymers can be applied as catalysts, if their physico-chemical properties are suitable for a particular chemical environment. Polymer matrices have been used as catalysts for several applications such as complex immobilization, in asymmetric reactions, water treatment, racemic separations, chelation with ions, dye fixations, etc. [30].

To synthesize an organic polymeric catalyst, two approaches can be utilized. In the first option, the monomer is functionalized to provide the catalytic activity, and afterwards, the polymerization reaction is carried out. Naturally, the chemical functionalization must not interfere in the reactivity of the potential polymerization groups. The second option is simply the reverse process of it, where polymerization takes first place, followed by a functionalization reaction performed in the polymer chains. In this case, one has to consider several polymeric properties that might lower the yield of the chemical functionalization, such as solubility of the polymeric matrix in the reaction medium, spatial hindrance due to the polymeric chains conformation, thermal and chemical resistance of the polymeric chains at specific conditions of the functionalization reaction, and others [31,32].

**Figure 4:**
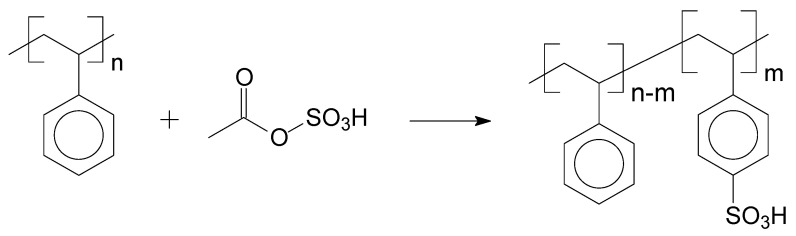
Sulfonation of PS with acetyl sulfate.

To provide the catalytic activity, one of the possible functionalization reactions for aromatic compounds is the sulfonation, which is a substitution reaction to bind a sulfonic group (SO_3_H) in the aromatic ring of a general chemical structure. This substitution occurs in the majority of the cases, forming a bond between the sulfonic group and a carbon of the aromatic ring structure or, in specific situations, substitution takes place in nitrogen atoms of the heterogeneous aromatic ring. Sulfonation agents can be sulphuric acid (H_2_SO_4_), sulphur trioxide (SO_3_), or their derivate compounds, for example, acyl or alkyl sulphates (C_2_H_6_SO_4_), chlorosulfonic acid (HClO_3_S), sulphur dioxide (SO_2_), sulphites (R_2_SO_3_), and hydrogen sulphide (H_2_S). The sulfonation mechanism of low Dalton mass molecules can be extrapolated for high Dalton mass molecules (polymers). The first report about sulfonation reaction with high Dalton mass compounds was published before the Second World War, with a sulfonated styrene matrix [33,34]. An example of aromatic polymer sulfonation is presented in Figure 4, which is the sulfonation of polystyrene (PS) [31]:

In Figure 1, in the sulfonated polymer, the sulfonic group can easily donate protons to the reaction medium due to the stabilization of the electrons pair by the oxygens and sulphur atom. The sulfonation technique is well reported in the literature for several applications, being used for example as ion exchange resins [35], ion exchange membranes [36,37], electrolyte membranes for fuel cells [38], and others.

## 2. Materials and Methods

### 2.1. Materials

4-hydroxybenzoic acid, 2-hydroxybenzoic acid, 2,4-dihydroxybenzoic acid, zinc acetate, acetic anhydride, methanol, tetrahydrofuran (THF, suitable for HPLC, ≥99.9% and inhibitor-free), potassium bromide, chloroform, sulfuric acid, ethyl ester, sodium hydroxide (NaOH), hydrochloric acid (HCl), phenolphthalein, and deuterated methanol (MeOd) for NMR, were acquired from Sigma-Aldrich (Darmstadt, Germany) and were used as received.

### 2.2. Polyesters Synthesis and Sulfonation Reaction

Polyesters were synthesized based in the synthesis described by DONG et al. (2001) [39], which proposes the formation of polyesters through a trans-acylation reaction using a Lewis acid catalyst. The methodology was adapted under two experimental procedures:

#### 2.2.1. Experimental Procedure 1 (EP-1)

In a 100-mL two-neck flask 6.90 g (50 mmol) of the hydroxy acid was added, afterwards was added 20 mg (0.09 mmol) of zinc acetate and 30 mL of acetic anhydride as solvent. A reflux condenser had been coupled in the central neck flask and a nitrogen flux was connected to the side neck flask. After that, the system was purged with nitrogen for 30 min before starting the reaction. In parallel, the reaction system was immersed in a heating bath stabilized at 120 °C. After temperature stabilization, the reaction took place over 4 h. After the reaction period, the heating was turned off and the system was left to be cooled to room temperature. In the meantime, the system was attached to a vacuum pump. The heating bath was now heated to 75 °C, under reduced pressure, utilizing the vacuum pump. After 2 h, the reaction system was disconnected from the vacuum pump, and the nitrogen flow was inserted again and heated to 250 °C for 2 h. Afterwards, the system was left to be cooled to room temperature. The obtained product was washed with methanol (3 times with 30 mL) and dried in an oven at 60 °C, until constant weight.

#### 2.2.2. Experimental Procedure 2 (EP-2)

In a 100-mL two-neck flask 6.90 g (50 mmol) of the hydroxy acid was added, 20 mg (0.09 mmol) of zinc acetate and 30 mL of acetic anhydride as solvent. A Dean-stark apparatus was attached to the central neck of the flask, and in the other neck, nitrogen flow was connected and left for purging the system during 30 min before starting the reaction. Afterwards, the system was immersed in a heating bath at 250 °C and it remained for 2 h. After removing the acetic anhydride in excess and the reaction sub-product (acetic acid) through the Dean-stark apparatus, the system was attached to a vacuum pump and reduced pressure was applied for 2 h. The obtained product was washed with methanol (3 times with 30 mL) and dried in an oven at 60 °C, until a constant weight was achieved.

After polymerization and characterization of the chemicals described above, oligomers with an average molar mass of 5500 g·mol^−1^ were obtained. Oligomers were named as follows: poly(4-hydroxybenzoic acid), poly(2-hydroxybenzoic acid), and poly(2,4-dihydroxybenzoic acid) and used as precursors to perform functionalization through a sulfonation reaction.

#### 2.2.3. Sulfonation Reaction Procedure

In a 50-mL flask, the polymer (1.0 g; 7.35 mmol of repetitive units) was added and 10 mL of chloroform. In parallel, the sulfonation solution was prepared as it follows: in a 25-mL beaker immersed in an ice bath, a solution containing 0.41 mL (7.35 mmol) of sulfuric acid and 0.97 mL (9.55 mmol) of acetic anhydride (30 mol% of excess) was prepared. While still in the ice bath, and after reaching an approximately constant temperature of 5 °C in the sulfonation solution, it was added to the 100-mL flask containing the solubilized polymer. The system was held under magnetic stirring at 600 rpm for 24 h and at room temperature. After the reaction time, the solid product was separated by decantation and washed several times with deionized water until achieving a neutral pH, and afterwards it was dried at 55 °C until a constant weight was achieved.

### 2.3. Characterization Methods

Characterization of the synthesized polyesters was carried out through infrared spectroscopy (FTIR), Gel Permeation Chromatography (GPC), and Thermogravimetric Analysis (TGA). After sulfonation, oligomers were characterized as well as by NMR, GPC, and titration.

#### 2.3.1. Fourier-Transform Infrared Spectroscopy (FTIR)

FTIR spectra were acquired in a spectrometer Bomem of Hartmann and Braun, model B-100 (ABB, Zürich, Switzerland). The samples were prepared in the solid phase, following the simple procedure of weighting 5 mg of the sample and 150 mg of potassium bromide. Both were homogenized with a pestle and mortar. The mixture was pressurized in a hydraulic press applying 18 Tonnes of pressure, forming tablets of 10-mm diameter by approximately 2- to 3-mm thickness. The spectra were obtained in a range between 500 and 4000 cm^−1^, with resolution of 4 cm^−1^ and 32 scans accumulation.

#### 2.3.2. Gel Permeation Chromatography (GPC)

GPC chromatograms were performed in a model 1515 (Waters, Milford, CT, USA), with two polystyrene-divinylbenzene (PSDVB) columns coupled in series (TSK Gel 1000 e Styragel 100), oven (PE003-HPLC/GPC-Waters) and a refraction index detector (Waters 2414). All samples were prepared with approximately a 5 mg·mL^−1^ concentration, using THF suitable for HPLC as the solvent. Control parameters were: flow of 0.8 mL·min^−1^, pressure of 860–1100 Bar, and the oven and detector at an isothermal condition of 40 °C.

#### 2.3.3. Thermogravimetric Analysis (TGA)

Samples were previously dried in a vacuum oven at 50 °C for 12 h. TGA curves were obtained in a model STA 409 C (NETZSC, Selb, Germany). Approximately 20 mg of the samples were weighted in a crucible for TGA analysis. Experiments were performed with a heating ramp of 10 °C·min^−1^, under oxidative atmosphere (synthetic air) and temperature range from 25 to 500 °C.

#### 2.3.4. Nuclear Magnetic Resonance of Hydrogen

^1^H NMR spectra were acquired in a device model AC 200, 200 MHz (Bruker, Karlsruhe, Germany). All samples were prepared as follows: approximately 40 mg of Oligocat was weighed in a 1.5 mL vial, and afterwards solubilized in circa 1 mL of deuterated methanol with tetramethyl silane (TMS) as reference. Finally, the solution was transferred to an NMR tube and analysed.

#### 2.3.5. Qualitative Solubility Evaluation

In a tube test with screw cap, was inserted 3.0 mL of solvent. 3%m·V^−1^ of oligocat was previously weighed and added to the tube test. A small magnet was inserted into the solution and the tube test was tightly closed to avoid solvent evaporation. The solution remained stirring (300 rpm) at room temperature for 12 h (overnight). Afterwards, the solubility of the samples was visually evaluated.

#### 2.3.6. Acidity Quantification through Titration

In a tube with a screw cap, 60 mg of Oligocat was weighted and 10.0 mL of standardized solution of NaOH (0.1 mol·L^−1^) was added. The mixture was closed to void solvent evaporation and kept under room temperature and magnetic stirring (600 rpm) for 24 h. Afterwards, 300 µL of phenolphthalein (solution 3% (m·V^−1^) in ethanol) was added to the Oligocat solution and titration was realized with a standardized HCl solution (0.05 mol·L^−1^)

## 3. Results and Discussion

### 3.1. Mass Polymerization of the Aromatic Acid Compounds

The compounds 4-hydroxybenzoic, 2-hydroxybenzoic, and 2,4-hydroxybenzoic acids, when submitted to the described reaction conditions, undergo via acetylation followed by mass polymerization reaction, catalysed by zinc acetate [39]. An intermediary compound is formed before the growth of the polymeric chains. During the course of the reaction, the monomers (aromatic acid compounds) are acetylated in the phenol groups and afterwards the transacylation between esters and acid groups takes place, generating the polymer chains and acetic acid as the side product.

**Figure 5:**
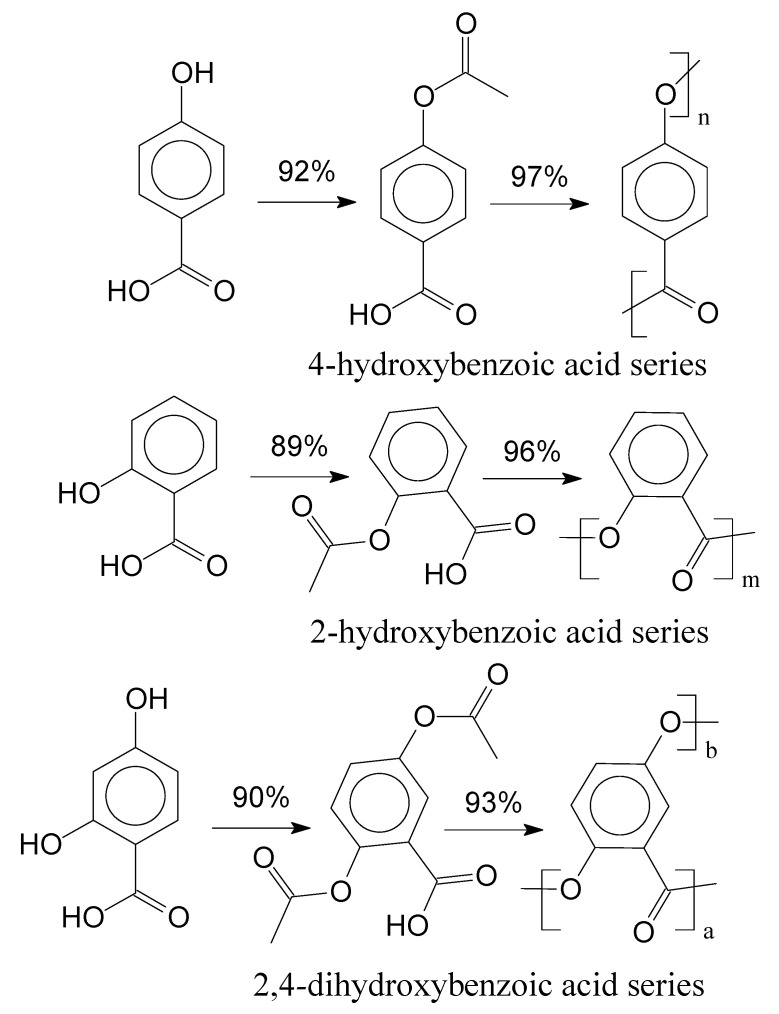
Acetylation and polymerization of aromatic acids and their yields.

During acetylation and polymerization reaction steps, samples were retrieved from EP-1 and EP-2. In EP-2, it was only possible to evaluate the final product. Interestingly, during the polymerization reaction the absence of the nitrogen atmosphere resulted in dark, vitrified, and brittle polymers, for both experimental procedures. Under Nitrogen atmosphere, polymers presented Heller colours and were less brittle. Dong et al. (2001) suggests that the dark coloration indicates a higher amount of degradation products or either crosslinking reaction between the polymer chains [39]. Figure 5 presents the reaction for each studied aromatic acid and their yields for EP-1.

**Figure 6:**
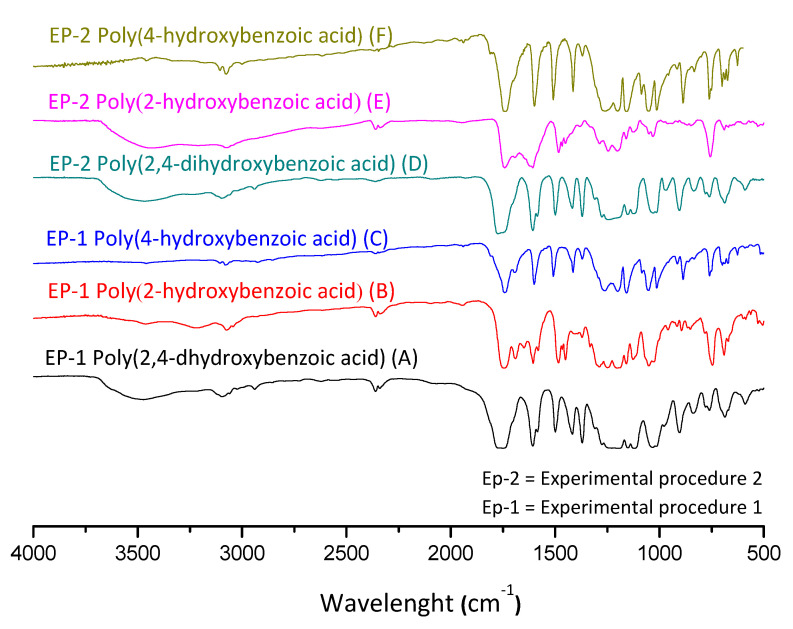
FTIR spectra of the polyesters obtained from experimental procedures 1 and 2.

In Figure 5, yields were calculated by comparing the molar quantity of the precursor with the obtained molar quantities of the products for each reaction step. Afterwards, the final products of the experimental procedures were evaluated through FTIR spectroscopy, which are presented in Figure 6.

**Figure 7:**
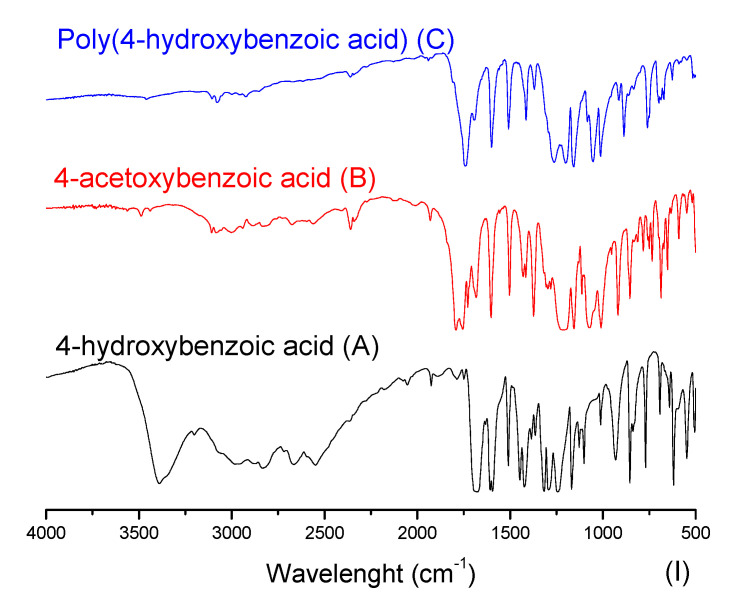

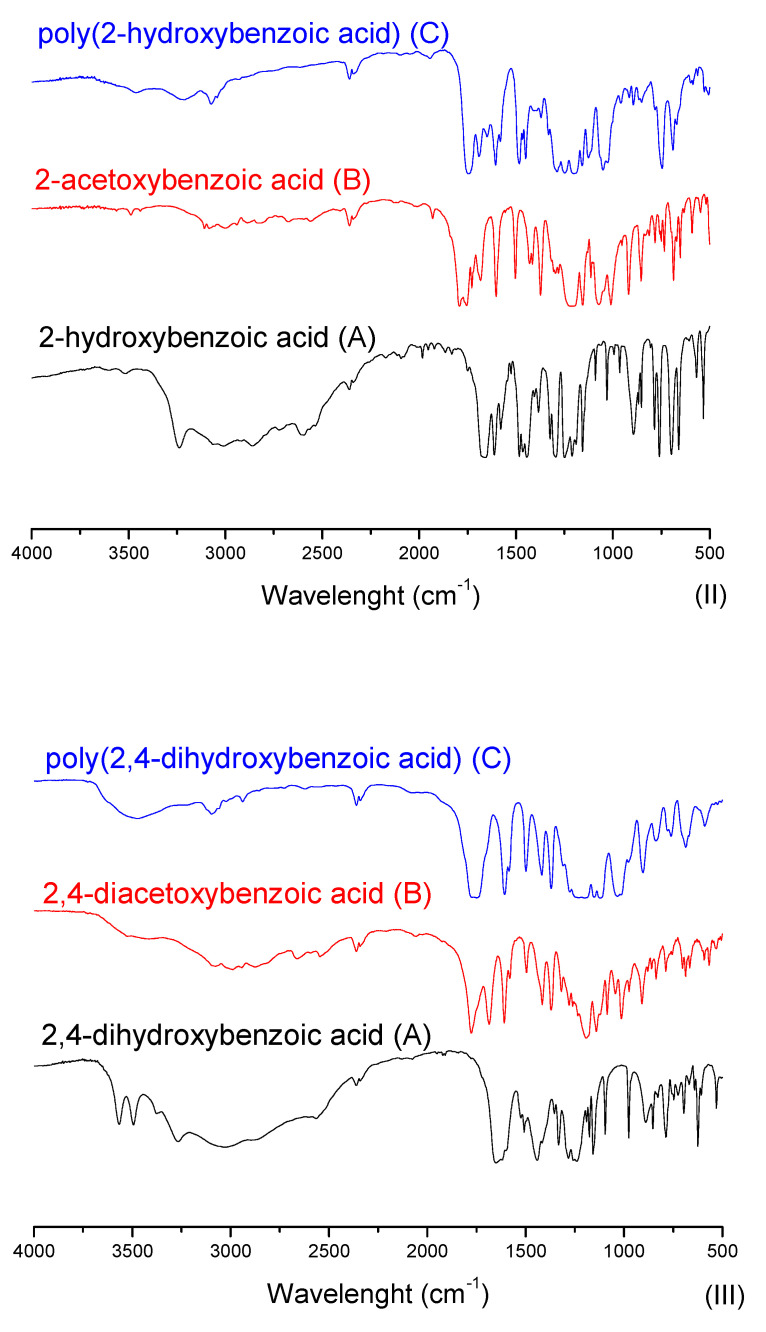
Aromatic acids, acetylated and polymer products FTIR spectra.

In Figure 6, one can note the similar profile of the final products for both experimental procedures, where the yields presented similar values. In order to better explore the reaction system, the discussion of the characterization is handled based on the EP-1, in which it is possible to evaluate the intermediate product. Figure 7 presents FTIR spectra for the obtained acetylated and polymer products for each studied aromatic acid.

In Figure 7, at the aromatic acid precursors (label A) the 3300 cm^−1^ stretching is typically assigned to axial bound deformation of hydrogen and oxygen in phenols. Once the phenol group is acetylated (intermediary products of label B), the intensity of 3300 cm^−1^ stretching is significantly reduced. Still, one can observe the 1197 cm^−1^ stretching, which can be compared between the aromatic acid precursors (label A) and their acetylated versions (label B). This stretching is assigned to the bound deformation of carbon and oxygen, typically found in carbonyl, ester, and lactone groups. At the carbonyl region, from 1680 to 1715 cm^−1^, at the acetylated compounds (label B), the stretching in this region is expected to be shifted to the left if compared with aromatic acid precursors (label A), evidencing the acetylation reaction. Interestingly, a certain number of the acid groups seem to have also reacted, as the spectra with labels B and C do not present significant stretching at hydroxyl groups. The stretching assigned to carbonyl groups from acid is significantly shifted to the left. This effect can be attributed to the formation of the anhydride group. That said, one can observe in the polyesters (label C) compared with acetylated compounds (label B), where a slight shift to the right (1690 cm^−1^) in the stretching assigned to the carbonyl groups is observed. This effect indicates the consumption of anhydride groups, being polymerized and remaining as the ester group in the final product [40,41]. Furthermore, in Figure 7, spectra III presents the FTIR from 2,4-dihydroxybenzoic acid and its products, which have a singularity. Their molecular structure has three reactive active groups that can be polymerized (two phenol groups and one acid). Nevertheless, in this polymerization technique, the acid group may participate in the course of the reaction, which leads to one free phenol group after polymerization.

**Figure 8:**
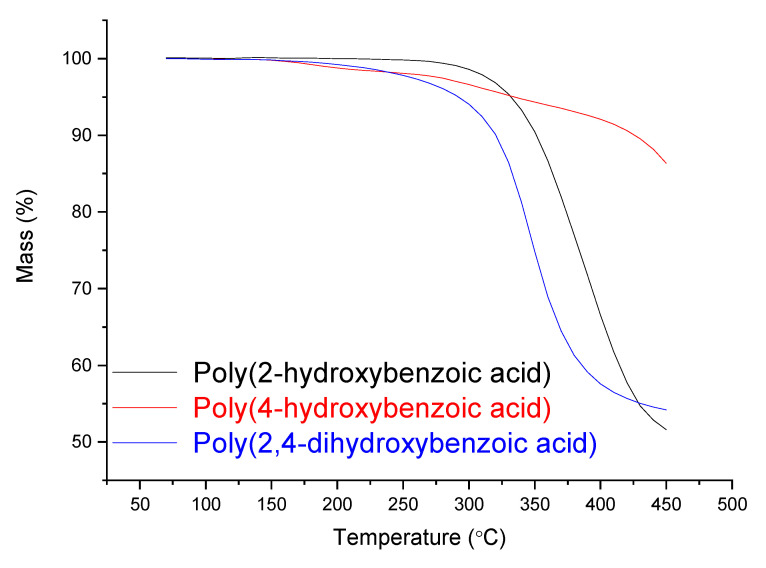
TGA curves of the oligomers.

In the Supplementary Materials, Tables S1 and S2 present the solubility evaluation the non-sulfonated and sulfonated polymers (Oligocat). Observing Table S2, one can see that only products from 2,4-dihydroxybenzoic acid and originated from methodology 1 are completely soluble in THF. Thus, these products were investigated regarding the size and distribution of the polymer chains. Through GPC, one could characterize its main properties, presenting Mw = 5357 g·mol^−1^ and Mn = 3909 g·mol^−1^, presuming oligomer compounds. The polydispersity was 1.37, a reasonable value due to the mass polymerization technique. Afterwards, the oligomers were investigated regarding thermal stability. Figure 8 presents the TGA curves for the obtained oligomer products.

In Figure 8, one can see enough thermal stability for all three investigated oligomers, where until 280 °C no significant mass loss is observed. Up from approximately 300 °C, the oligomers start lose their masses constantly. Nevertheless, oligomer poly(4-hydroxybenzoic acid) presented even higher thermal resistance until 450 °C, with less than 15% mass loss.

### 3.2. Sulfonation of the Oligomers

**Figure 9:**
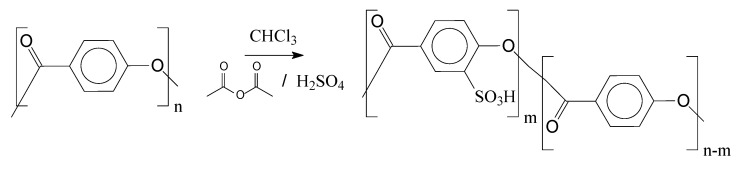
Poly(4-hydroxybenzoic acid) sulfonation.

Figure 9 depicts the proposed reaction to insert the catalytic groups (sulfonic groups) in the oligomeric chains, using as example the poly(4-hydroxybenzoic acid) as an oligomer precursor.

**Figure 10:**
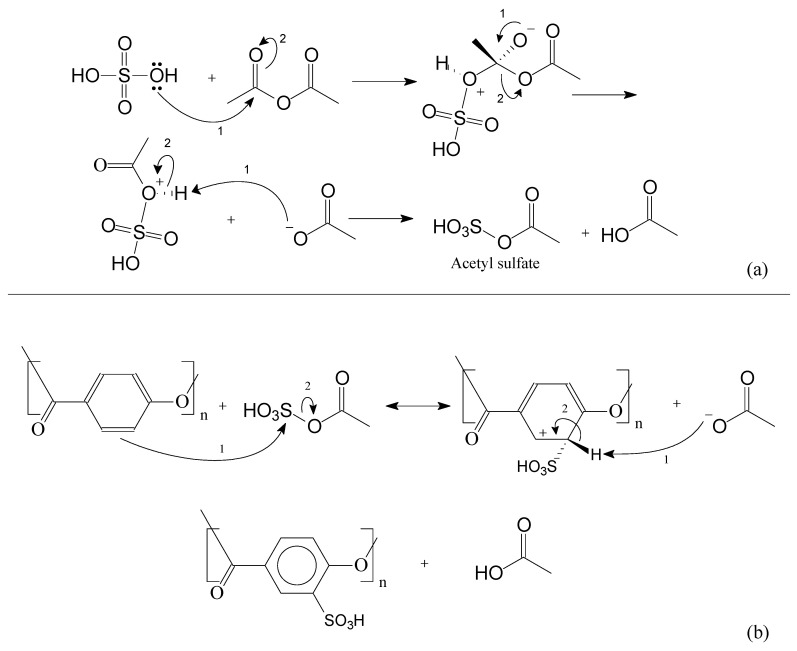
Sulfonation reaction. In (**a**): the acetyl sulfate mechanism and in (**b**) the aromatic electrophilic substitution mechanism.

During the course of the sulfonation reaction, acetyl sulfate is generated, which is the active compound to sulfonate the oligomers. Acetyl sulfate is known as having lower reactivity if compared with sulfuric acid. This strategy is applied to avoid excessive oxidation caused by sulfuric acid. Following Figure 9, in Figure 10 is presented the main mechanism of the acetyl sulfate formation. Afterwards, one can observe the sulfonation reaction via the aromatic electrophilic substitution mechanism.

After sulfonation, the oligomers were characterized regarding the substitution ratio of the sulphonic groups, which determines the catalytic activity for our material. The number of sulphonic groups was determined through titration, ^1^H NMR, and GPC techniques. It is important to observe that one of the sulfonated oligomers, the poly(4-hydroxybenzoic acid), was insoluble in several chemical environments, which precluded its characterization. By titration technique, results from 35 to 50% of substitution were found, as Table 1 presents.

**Figure 11:**
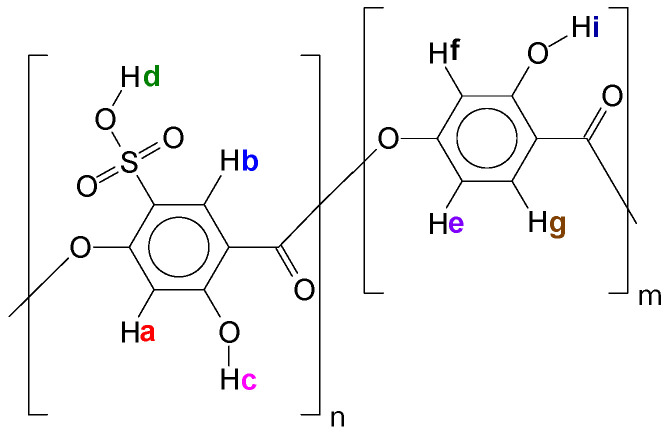
Poly(2,4-dihydroxy-5-sulfobenzoic acid) molecular structure with labeled hydrogens.

In Table 1, one can see an expressive deviation for SO_3_H content. Although the titration technique is the cheapest and fastest method to quantify Oligocat catalytic activity, this procedure might have interferences that can lower the quantification. As Oligocat is insoluble in aqueous solution, the likelihood of not reacting all active sulfonic groups is prone to happen. In order to compare the results, the sulfonic content was investigated using ^1^H NMR and GPC. Initially, to better understand the Oligocat NMR spectroscopic behaviour, Figure 11 presents the molecular structure of poly(2,4-dihydroxy-5-sulfobenzoic acid) with labeled hydrogens.

In Figure 11, it is possible to observe one of the Oligocat molecular structures, composed of two different repetitive units. Before the spectra interpretation, firstly, it is discussed here how hydrogens might interact in this chemical environment. Hydrogens Hi, Hc, and Hd can easily exchange with the deuterium from solvent (Methanol D4). Therefore, one might observe no signal for these hydrogens. Ha couples with Hb, however, as this coupling has a long distance (J = 0~1 Hz) commonly one will observe a singlet (s) signal. In Hf, a meta coupling with He is expected. Still Hf could couples with Hg; nevertheless, the coupling constant is not strong enough to deploy the peaks at the conditions of this ^1^H NMR experiment. He will have two possible couplings, ortho and meta, where it can be generally observed coupling constants of 6 to 10 Hz and 1 to 3 Hz, respectively. Therefore, He has a double doublet (dd). Finally, in Hg, there is the coupling in ortho with He, characterizing a doublet (d) signal.

**Figure 12:**
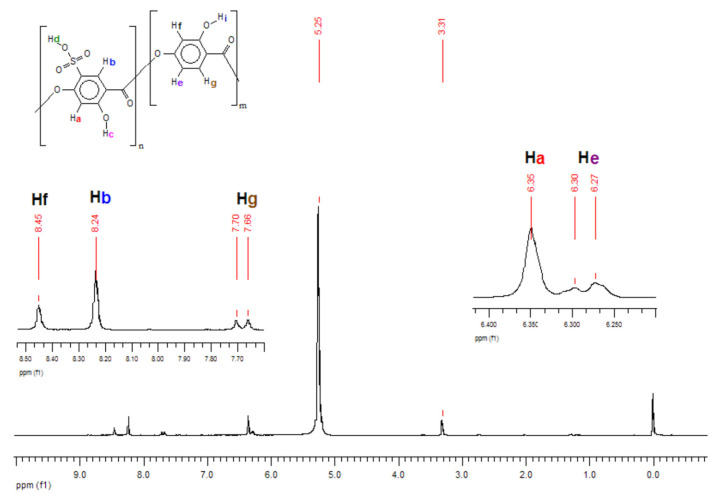
^1^H NMR spectra of poly(2,4-dihydroxy-5-sulfobenzoic acid).

Observing the spectra in Figure 12, the peaks in 5.25 and 3.31 ppm are assigned to the residual hydrogens of methanol present in the solvent CD_3_OD. Since hydrogens Hc, Hi and mainly Hd are acid labile, as discussed, exchanging them with deuterium from solvent is expected, and this effect will also increase the methanol peak intensity. Side products can be investigated in the region of 1.50 to 2.50 ppm, where it was observed weak peaks, assigned to acetic anhydride (2.23 ppm) and acetic acid (2.13 ppm). Hydrogens Ha and Hb were assigned to singlets in 6.34 and 8.23 ppm, respectively. For Hg, a well-defined doublet in 7.68 ppm with a coupling constant of 8.6 Hz was assigned. He should present a double doublet peak with constants of 6 to 10 Hz (ortho) and 1 to 3 Hz (meta) couplings. Nevertheless, was observed only typical ortho coupling for this hydrogen (6.28 ppm, 7.7 Hz). As there are two spin systems in the structure, the integration values for hydrogens of non-sulfonated and sulfonated repetitive units were compared and, a ratio of approximately 1:2 was found, indicating sulfonation of 67% in mass.

The sulfonation content was also investigated through GPC, were the averaged molar mass was compared before and after the sulfonation reaction. For this simple calculation, it was considered only one insertion of the sulfonic group per aromatic ring. Table 2 summarizes the sulfonic content values calculated within the three characterization techniques.

**Figure 13:**
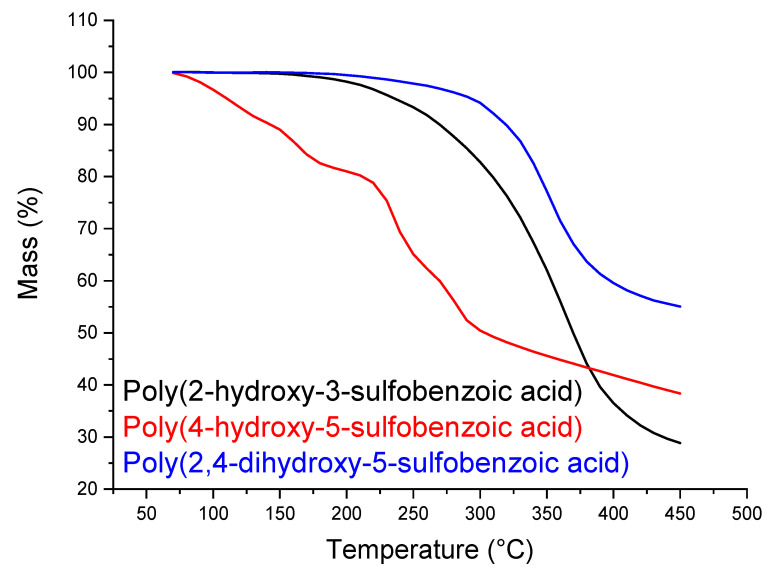
Thermogravimetric analysis of the sulfonated oligomers (Oligocat).

In Table 2, one can see similar results of SO_3_H content by ^1^H NMR and GPC characterization techniques. Titration, as discussed, might not be a very efficient technique to calculate the Oligocat substitution rate. Figure 13 presents the thermal stability of the sulfonated oligomers (Oligocat) catalysts.

In Figure 13, poly(2-hydroxy-5-sulfobenzoic acid) initiates the mass loss at 220 °C until 270 °C, which can be assigned to the separation of the sulfonic groups from the polymeric chain and, at 360 °C, the polymeric chain cleavage occurs [42]. TGA curve of poly(4-hydroxy-5-sulfobenzoic acid) presented less thermal stability. From 120 to 160 °C occurs the sulfonic groups degradation and, at 240 °C, the cleavage of the polymeric chains most probably occurs. Poly(2,4-dihydroxy-5-sulfobenzoic acid) presented a similar thermal profile as poly(2-hydroxy-5-sulfobenzoic acid). Nevertheless, a considerable mass is remained, which can indicate better stability.

Preliminarily, Oligocat was evaluated as a Brønsted–Lowry acid catalyst for biodiesel production from second generation feedstock. A promising conversion rate above 96.5 wt.% was achieved applying tallow swine as the TAG source, and methanol as the alkylation agent. Naturally, this high conversion rate was an outcome after reaction parameters optimization, where a detailed investigation is presented by Vlnieska et al. (2021) [3].

## 4. Conclusions

The synthesis and characterization of a catalyst for biodiesel production, named Oligocat was presented. Oligocat synthesis can be described as having two main steps: first, the mass polymerization of aromatic acids, and second, the polymeric chains functionalization through sulfonation technique. Low molar masses of 5357 g·mol^−1^ (Mw) and 3909 g·mol^−1^ (Mn) were obtained (poly(2,4-dihydroxybenzoic acid)), classifying the compounds as oligomers. Relying on GPC and ^1^H NMR characterization techniques, a high substitution rate of approximately 70 mol% was obtained for the sulfonic groups. The sulfonic groups provide the catalytic property to Oligocat. Thermal resistance of Oligocat was investigated and TGA indicated that chemical environments until 175 °C can be safely applied for two of the Oligocat catalysts, causing no degradation of the oligomeric matrixes. Oligocat is a Brønsted–Lowry acid catalyst for biodiesel production.

## Figures and Tables

**Table 1:** Oligomers SO_3_H content determined by titration.

Compound	SO_3_H Content (mmol·g_polymer_^−1^)	SO_3_H Substitution (mol%)
P4S ^a^	4.05	48.6
P2S ^a^	3.88	46.6
P24S ^b^	2.85	38.0

P4S: Poly(4-hydroxy-3-sulfobenzoic acid). P2S: Poly(2-hydroxy-5-sulfobenzoic acid). P24S: Poly(2,4-dihydroxy-5-sulfobenzoic acid). ^a^: 1 g polymer = 8.326 mmol of repetitive units. ^b^: 1 g polymer = 7.407 mmol of repetitive units.

**Table 2:** Calculated sulfonation content of poly(2,4-dihydroxy-5-sulfobenzoic acid) through titration, GPC, and ^1^H NMR.

Technique	SO_3_H Content (mmol·g_polymer_^−1^)	SO_3_H Substitution (mol%)
^1^H NMR	4.92	67.3
GPC	5.06	68.9
Titration	2.85	38.0

## Supplementary Materials

The following supporting information can be downloaded at: https://www.mdpi.com/article/10.3390/polym14010019/s1, Table S1: non-sulfonated oligoesters solubility evaluation (qualitative study); Table S2: sulfonated oligoesters solubility evaluation (qualitative study).
