# Supplementary material for: Synthesis and Chemical Functionalization of Pseudo-Homogeneous Catalysts for Biodiesel Production—Oligocat
Source: Polymers (Basel). 2021 Dec 22;14(1):19. doi: 10.3390/polym14010019 (PMC8747319; doi:10.3390/polym14010019)
Supplement: Supplementary file 1 [file polymers-14-00019-s001.zip › polymers-1503017-supplementary.pdf]

polymers MDPI

Supplementary material:

Table S1: non-sulfonated oligoesters solubility evaluation (qualitative study).

**Metodology 01**	**MeOH**	**$CHCl_3$**	**Hex**	**DMF**	**Acetone**	**H_2O**	**DCM**	**EtOH**	**THF**
Poly(4-hydroxybenzoic acid)	n	n	n	n	n	n	n	n	n
Poly(2-hydroxybenzoic acid)	n	s	n	s	s	n	s	n	s
Poly(2,4-dihydroxybenzoic acid)	n	s	n	s	s	n	s	n	s
**Metodologia 02**	**MeOH**	**CHCl3**	**Hex**	**DMF**	**Acetone**	**H_2O**	**DCM**	**EtOH**	**THF**
Poly(4-hydroxybenzoic acid)	n	n	n	n	n	n	n	n	n
Poly(2-hydroxybenzoic acid)	n	s	n	s	p(m)	n	s	n	p(m)
Poly(2,4-dihydroxybenzoic acid)	n	n	n	p(l)	n	n	n	n	p(m)

n = non-soluble
s = soluble
p(m) = partially much soluble
p(l) = partially less soluble

MeOH = methanol; $CHCl_3$ = chloroform; Hex = hexane; DMF = dimethylformamide; H_2O = water; DCM = dichloromethane ; EtOH = ethanol; THF = tetrahydrofuran.

Table S2: sulfonated oligoesters solubility evaluation (qualitative study).

**Metodologia 01**	**MeOH**	**CHCl3**	**Hex**	**DMF**	**Acetona**	**H_2O**	**DCM**	**EtOH**	**THF**
Poly(4-hydroxi-3-sulfo-benzoic acid)	n	n	n	p(m)	p(l)	n	n	p(l)	n
Poly(2-hydroxi-5-sulfo-benzoic acid)	p(m)	p(m)	n	s	s	n	p(m)	p(m)	p(m)
Poly(2,4-dihydroxi-5-sulfo-benzoic acid)	n	s	n	s	s	n	s	n	s
**Metodologia 02**	**MeOH**	**CHCl3**	**Hex**	**DMF**	**Acetona**	**H_2O**	**DCM**	**EtOH**	**THF**
Poly(4-hydroxi-3-sulfo-benzoic acid)	p(l)	p(l)	n	p(m)	n	n	n	p(l)	p(m)
Poly(2-hydroxi-5-sulfo-benzoic acid)	p(m)	p(m)	n	sim	p(m)	n	s	p(m)	p(m)
Poly(2,4-dihydroxi-5-sulfo-benzoic acid)	p(l)	n	n	p(m)	p(l)	n	n	p(m)	p(l)

n = non-soluble
s = soluble
p(m) = partially much soluble
p(l) = partially less soluble

MeOH = methanol; $CHCl_3$ = chloroform; Hex = hexane; DMF = dimethylformamide; H_2O = water; DCM = dichloromethane ; EtOH = ethanol; THF = tetrahydrofuran.
